# Social reintegration of women after obstetric fistula surgery: Evidence from a longitudinal multilevel mixed‐effects study in Zambia

**DOI:** 10.1111/aogs.70296

**Published:** 2026-06-23

**Authors:** Sianga Mutola, Bwalya Magawa Chomba, Nawi Ng, Menda M. Dhally, Valérie R. Louis, Lowery Wilson Michael

**Affiliations:** ^1^ Heidelberg Institute of Global Health, Faculty of Medicine and University Hospital Heidelberg University Heidelberg Germany; ^2^ Zambia Fistula Foundation Treatment Network Mansa Zambia; ^3^ School of Public Health and Community Medicine, Institute of Medicine, Sahlgrenska Academy University of Gothenburg Gothenburg Sweden; ^4^ School of Postgraduate Studies University of Lusaka Lusaka Zambia

**Keywords:** continence, longitudinal study, obstetric fistula, social reintegration, Zambia

## Abstract

**Introduction:**

Obstetric fistula remains a persistent maternal health challenge in sub‐Saharan Africa, severely impairing women's physical, social, and psychological well‐being. Although surgical repair restores continence, less is known about the factors influencing women's social reintegration after treatment. This study examined the association of continence status and follow‐up time on social reintegration among women who underwent obstetric fistula repair in Zambia.

**Material and Methods:**

This prospective cohort study included 2172 women who underwent fistula repair between 2017 and 2023 at eight hospitals across Zambia. Multilevel mixed‐effects models were used to assess the effects of continence and follow‐up time on a composite social reintegration score (0–100), derived from the five domains using principal component factor analysis (eigenvalue = 4.02) and demonstrating internal consistency (Cronbach's α = 0.94), while adjusting for baseline covariates.

**Results:**

Among all participants, 86.3% achieved continence, whereas 13.7% remained incontinent at discharge. The null model yielded negligible between‐patient variance (intraclass correlation coefficient, ICC <0.001). In the fully adjusted model, continent women had higher reintegration scores than their incontinent counterparts (*β* = 38.62; 95% CI: 36.10–41.14). Social reintegration improved substantially within the first 3 months after surgery (*β* = 10.83; 95% CI: 8.23–13.43), and remained stable at 6 months (*β* = 9.34; 95% CI: 6.70–11.98), and 12 months (*β* = 9.66; 95% CI: 6.97–12.34). Interaction analyses indicated that although continent women consistently reported higher social reintegration scores, the difference between continent and incontinent women narrowed over time, reflecting gradual improvements in social reintegration among incontinent women (*β* = −4.85; 95% CI: −6.30 to −3.40).

**Conclusions:**

Restoration of continence was the strongest predictor of improved social reintegration following fistula repair. However, the largest social reintegration improvement was observed among incontinent women within the first 3 months after surgery. These findings highlight the importance of combining high‐quality surgical care with structured early postoperative follow‐up and reintegration support to sustain recovery among women affected by obstetric fistula, regardless of the surgical repair outcome.

## Graphical abstract

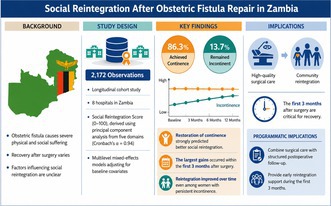

**Abbreviations**
FFTN: Fistula Foundation Treatment Network
ICC: intraclass correlation coefficient
ICF: International Classification of Functioning, Disability and Health
RVF: rectovaginal fistula
VVF: vesicovaginal fistula
WHOQOL‐BREF: World Health Organization Quality of Life Brief Questionnaire

**Key message**

Restoration of continence predicts social reintegration after obstetric fistula repair. Among incontinent women, the greatest improvements occur within the first 3 months, with gradual gains over time. Early postoperative support is critical to sustain recovery.

## 1 INTRODUCTION

Obstetric fistula is an abnormal communication between the vagina and bladder (vesicovaginal fistula) or rectum (rectovaginal fistula), most commonly resulting from prolonged obstructed labor that causes tissue necrosis and subsequent urinary and/or fecal incontinence.^1^, ^2^

Although obstetric fistula and other complications of prolonged obstructed labor have been substantially reduced in high‐income countries,^3^, ^4^ they remain a major challenge in resource‐constrained regions, particularly sub‐Saharan Africa (SSA).^1^, ^4^, ^5^ Recent estimates suggest that approximately 457 000 women aged 15–64 years are living with obstetric fistula globally, with the burden disproportionately concentrated in SSA, where prevalence is estimated at 71 per 100 000 women, nearly twice that reported in Asia.^6^ In Zambia, national survey data from the 2024 Zambia Demographic and Health Survey indicate that 0.3% of women aged 15–49 years reported ever experiencing fistula symptoms.^7^

Obstetric fistula remains strongly associated with limited access to timely and quality maternal healthcare, including emergency comprehensive obstetric care, particularly among women in rural settings.^5^, ^8^, ^9^, ^10^, ^11^

Once an obstetric fistula occurs, restoration of continence through catheterization or surgical repair is central to recovery and subsequent social reintegration.^12^ Evidence from sub‐Saharan Africa indicates that approximately 75–85% of women achieve continence following obstetric fistula repair, whereas 15–20% continue to experience persistent urinary incontinence despite successful closure.^13^ Consistent with these estimates, a recent nationwide study in Zambia reported an overall surgical success rate of 88.1%.^14^

In Zambia, access to fistula care has expanded through support from the Ministry of Health, the Fistula Foundation Treatment Network (FFTN), and the United Nations Population Fund, strengthening referral systems and access to surgical repair services.^5^, ^15^, ^16^ The Zambia FFTN has also strengthened community referral pathways for fistula screening and treatment through Primary Health Care structures.^16^

Women affected by obstetric fistula frequently experience physical, psychological, and social challenges, including stigma, discrimination, marital dissolution, and social isolation, which may persist even after surgical repair.^12^, ^17^ Consequently, restoration of continence alone may not guarantee successful reintegration into family and community life.^18^ Unfortunately, evidence from nationwide longitudinal studies examining social reintegration following obstetric fistula repair in Zambia remains limited. This study examined the association of post‐repair continence status, follow‐up time trends, and social reintegration among women who underwent obstetric fistula repair in Zambia.

## 2 MATERIAL AND METHODS

### 2.1 Study design and study site

This prospective cohort study used the Zambia FFTN clinical database, which included 2172 women who underwent surgical repair of obstetric fistulas from 2017 to 2023. The World Health Organization's International Classification of Functioning, Disability and Health (ICF) guided the conceptualization of social reintegration following fistula repair. In the ICF, health outcomes are conceptualized as resulting from interactions among body functions and structures, activity limitations, participation restrictions, and contextual factors.^19^ In this study, social reintegration was conceptualized as a functional participation outcome rather than a general measure of health‐related quality of life. Although the domains included in the social reintegration measure may overlap with broader quality‐of‐life constructs, they were used specifically to capture women's ability to resume social roles, economic activities, and interpersonal relationships following surgical repair, consistent with the participation domain of the International Classification of Functioning framework.^19^, ^20^

The participants, from all ten provinces of Zambia, received surgical repair in one of eight FFTN‐supported hospitals, each located in one of eight provinces: Chilenje Level 1 Hospital (Lusaka province), Monze Mission Hospital (Southern province), Kabwe Central Hospital (Central province), Solwezi General Hospital (North‐western province), St. Francis Mission Hospital (Eastern province), Mbala General Hospital (Northern province), Mansa General Hospital (Luapula province), and Chilonga Mission Hospital (Muchinga province). These facilities operate within a coordinated national fistula‐treatment network, using standardized clinical protocols and follow‐up procedures and a centralized database. While a patient can receive care from more than one treatment center, unique patient identifiers are used and linked across the treatment network to ensure that patients' entries are not duplicated across fistula treatment facilities. Furthermore, for this study, only records of one eligible surgical episode were retained per woman, and the dataset was structured longitudinally, with repeated social reintegration assessments recorded at discharge (baseline) and at 3, 6, and 12 months post‐surgery.

### 2.2 Data collection

**FIGURE 1:**
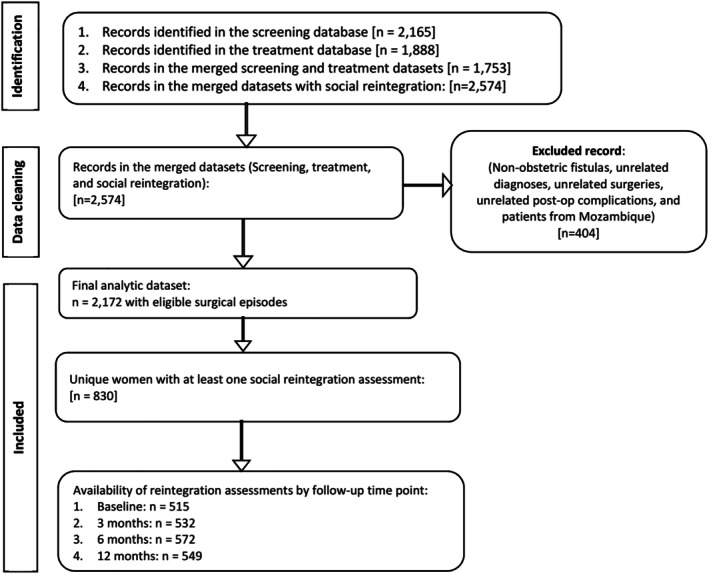
Flow diagram of participant selection and availability of social reintegration assessments in the obstetric fistula repair cohort, Zambia, 2017–2023.

Women who underwent obstetric fistula repair between 2017 and 2023 were identified from the central FFTN clinical database, which compiles standardized clinical and follow‐up records from fistula treatment hospitals across Zambia.^16^ Eligibility criteria were applied before analysis, and records that did not meet predefined clinical and data‐completeness requirements were excluded, as detailed in Figure 1.

The final analytical dataset included only women who underwent obstetric fistula repair and had at least one recorded social reintegration assessment. Furthermore, women who missed one or more scheduled follow‐up visits were not excluded from the analysis. However, women with no recorded reintegration data at any time point were excluded from the outcome analysis. This may introduce selection bias, as women who did not access care were not captured in the database, and those without a follow‐up record were excluded. Consequently, the findings may not fully be generalizable to all women living with obstetric fistula.

Patients were referred to treatment centers through community outreach activities, health facility referrals, and self‐presentation. Upon arrival at the treatment centers, trained clinicians collected standardized socio‐demographic and clinical information and confirmed the presence of a fistula using the Methylene Blue test. The full description of the patient identification and referral process is documented in Supplementary Material 1. The socio‐demographic and clinical data were recorded in hospital registers and subsequently entered into the centralized FFTN database.^16^ As part of routine postoperative follow‐up, patients were assessed at discharge (baseline) and at 3, 6, and 12 months after surgical repair using a structured social reintegration questionnaire developed and validated by the FFTN and Ministry of Health.^16^

The questionnaire, whose details are presented in Supplementary Material 2, included six items assessing continence status and patient‐reported functional and psychological outcomes, including the ability to socialize, the ability to work, satisfaction with life, satisfaction with health, and self‐esteem.^16^ These domains reflect participation and well‐being dimensions consistent with established health‐related frameworks, such as the World Health Organization Quality of Life (WHOQOL) Brief Questionnaire (WHOQOL‐BREF) and its application.^21^, ^22^

### 2.3 Measurements

#### 2.3.1 Independent variable

In this study, we used the fistula continence status (continent or incontinent) as the primary independent variable. The medical team examined all patients who underwent obstetric fistula surgical repair at discharge from the hospital, and then at 3, 6, and 12 months to assess their continence status. The surgeons confirmed fistula continence by physical examination and methylene blue testing. Therefore, we treated continence status as a time‐varying exposure in the longitudinal analysis in this study because it was reassessed at each follow‐up visit.

#### 2.3.2 Outcome variable

The primary outcome was the social reintegration score, a composite patient‐reported measure reflecting women's physical, social, and psychological recovery after obstetric fistula repair. The score was derived from five domains captured in the clinical follow‐up instrument used within the FFTN: ability to socialize with others, ability to work or perform usual activities, satisfaction with life, satisfaction with health status, and self‐esteem. Each domain was assessed at baseline and at 3, 6, and 12 months post‐surgery using a 5‐point Likert scale ranging from 1 (“very poor”) to 5 (“very good”).

For each participant and time point, responses across the five domains were averaged and linearly transformed to a 0–100 scale, with higher scores indicating better social reintegration. This scaling approach is consistent with methods used in similar widely applied instruments such as WHOQOL‐BREF and the SF‐36 Health Survey.^22^, ^23^

To support the construction of a composite social reintegration measure, we first assessed internal consistency across the five domains using Cronbach's alpha, which demonstrated high reliability across follow‐up periods (Cronbach's α = 0.94). We then conducted an exploratory factor analysis using principal component factor extraction to examine whether the domains reflected a common underlying construct. A one‐factor solution was retained (eigenvalue = 4.02), explaining 80.4% of the total variance. All five domains loaded strongly onto the latent construct (factor loadings: 0.78–0.94), supporting their aggregation into a composite social reintegration score. Predicted factor scores were generated for each observation and linearly transformed to a 0–100 scale, with higher scores indicating better social reintegration.

This approach allowed the score to reflect the shared variance across domains rather than assuming equal weighting of individual items. Although the measure was not formally validated as a standalone psychometric instrument, the selected domains were conceptually aligned with the participation component of the WHO International Classification of Functioning, Disability and Health framework and were intended to capture women's ability to resume social, functional, and interpersonal roles following fistula repair. An increase from 60 to 70 points indicates a shift toward better perceived ability to engage in social and economic activities, consistent with improvement across multiple dimensions of recovery following surgical repair.

#### 2.3.3 Baseline time‐invariant covariates

In our analysis, we included baseline factors assessed during patient screening and treatment, categorized as socio‐demographic characteristics, fistula characteristics, and clinical factors. The socio‐demographic factors included age, marital status, parity (number of live births),^24^ and the province of residence in Zambia. These baseline variables were included as covariates in the multivariable mixed‐effects models to adjust for potential confounding in the association between continence status and social reintegration. Age was categorized as <20, 20–34, and ≥35 years to reflect adolescent, peak reproductive age, and advanced maternal age groups.^25^

Marital status was dichotomized into not married and in union. The parity level was classified as 0, 1–3, and ≥4. The Zambian provinces where the patients lived included Eastern, Luapula, Lusaka, Central, Copperbelt, Western, Muchinga, Northern, North‐western, and Southern. Zambian provinces are generally divided into urban and rural.^26^

In this study, we grouped the provinces into geographic clusters based on geographic proximity, cultural similarities, and potential shared socioeconomic barriers, thereby enhancing the interpretability of the analysis. In this regard, Eastern and Luapula provinces were left to stand alone because each had slightly different social dynamics and cultural characteristics. The other provinces were clustered as follows: Central, Lusaka, Southern, and Western; Copperbelt and North‐western; and Northern and Muchinga.

The fistula characteristics included obstetric fistula type, Waaldijk's obstetric fistula classification,^3^ previous fistula repair, years lived with the fistula, and type of surgical procedure. Surgeons verified the anatomical diagnosis recorded in the database, including vesicovaginal fistula (VVF), rectovaginal fistula (RVF), or combined VVF and RVF. For analytical purposes, cases with RVF only and those with both VVF and RVF were grouped into a single category (RVF or both) to distinguish women with any rectovaginal involvement from those with isolated urinary fistula. This categorization reflects clinical evidence that fistulas involving the rectovaginal structure, whether isolated or combined with VVF, generally represent severe injury patterns and are associated with poorer surgical prognosis than isolated VVF.^27^, ^28^ The fistula type variable was therefore dichotomized as “VVF” and “RVF or both”.

The surgeons also recorded whether a woman had undergone a previous fistula repair before the current surgery. This variable was included as a binary covariate (previous repair: yes/no) in the analysis to account for potential differences in prognosis between primary and repeat repairs. Each record in the analytic dataset corresponds to a single surgical episode. Repeat repair attempts during the follow‐up period were not included as separate records.

Furthermore, we categorized the patient‐reported duration of time the women lived with the obstetric fistula as <1 year, 1–5 years, or >5 years. Lastly, the surgeons recorded the type of surgery conducted. These were generally related to the type of obstetric fistula, so we categorized all surgical repairs involving urinary tract structures as VVF. In contrast, “Others” included RVF and other types, such as perennial tears resulting from childbirth.

With respect to clinical factors, we evaluated patients' referral pathways, hospitals of care (treatment centers), surgeon‐reported surgical difficulty, and the presence of postoperative complications. We dichotomized the referral system into “volunteer and self” and “health and fistula foundation staff”. The hospitals were grouped by geographic proximity, as patients in those areas were likely to seek care at nearby hospitals, regardless of provincial boundaries. Therefore, the hospitals were clustered as follows: Kabwe, Monze, and Chilenje; Solwezi; Northern region hospitals (Mbala/Mansa/Chilonga); and St. Francis.

The operating surgeons recorded the surgical repair's difficulty as simple, intermediate, or difficult based on specific factors, including a narrow vaginal caliber (<2 cm), minimal residual anterior vaginal wall, the need for ureteric stenting, bilateral dense lateral contractures, the use of a combined abdominal and vaginal approach, and the presence of a recto‐vesicovaginal fistula.^29^ Finally, we categorized postoperative complications after fistula repair as absent or present, based on surgeons' records. All covariates were treated as time‐invariant variables in the analysis to control for confounding.

### 2.4 Statistical analysis

The FFTN database was recorded in Microsoft Excel and imported into Stata/SE 18 for Windows (StataCorp LLC, College Station, TX, USA) for cleaning and analysis in accordance with the inclusion and exclusion criteria shown in Figure 1. A detailed description of the data cleaning, missing data assessment, model specification, and sensitivity analyses is provided in Supplementary Material 3.

Each record represented one surgical episode for a woman. Exclusion criteria were applied at the record level before analysis, resulting in the exclusion of 404 records such as non‐obstetric fistula diagnoses (*n* = 82), unrelated surgical diagnoses (*n* = 153), non‐informative continence status at discharge (*n* = 134), procedures not relevant to obstetric fistula repair (*n* = 160), treatment outside Zambia (*n* = 10), non‐eligible postoperative complications (*n* = 19), and missing or non‐informative incontinence status in the reintegration questionnaire (*n* = 68). Because some records met more than one exclusion criterion, category counts exceed the total number excluded. Therefore, the final analytical cohort consisted of 2172 women who underwent obstetric fistula repair between 2017 and 2023. Descriptive statistics summarized participants' socio‐demographic and clinical characteristics as column proportions, while means and 95% confidence intervals (CI) were calculated for social reintegration domains across follow‐up periods. Multicollinearity among covariates was assessed before fitting multivariable models.

Approximately 33% of observations were complete, whereas 67% had incomplete information across one or more variables, primarily for fistula classification (35%) and social reintegration score (22%). Missingness partly reflected irregular attendance at routine follow‐up visits, including non‐monotone follow‐up patterns in which women missed the interim visits but returned for later assessments. Missingness appeared related to observed patient, clinical, and follow‐up characteristics, supporting the plausibility of the Missing at Random (MAR) mechanism.^30^, ^31^ Therefore, Multiple Imputation by Chained Equations (MICE) with 20 imputations was applied to retain incomplete observations and reduce potential bias.^30^, ^32^ The imputation included the social reintegration score, continence status, follow‐up time, treatment facility, and all analytical covariates. Complete‐case sensitivity analyses using the same model specifications were additionally conducted.

Multilevel mixed‐effects linear regression models were fitted to examine the association of continence status and follow‐up time with social reintegration. Repeated measurements at baseline, 3, 6, and 12 months were nested within women. Therefore, patient‐level random intercepts were included to account for within‐person correlation across repeated observations.^33^ Model assumptions were assessed using residual diagnostics, with no major deviations observed.

An intercept‐only (null) model was fitted to estimate variance components and the intraclass correlation coefficient (ICC), calculated as *τ* ^2^/(*τ* ^2^ + *σ* ^2^), where *τ* ^2^ represents the between‐patient variance and *σ* ^2^ the residual variance. The ICC was <0.001, indicating minimal between‐women variation in social reintegration scores and suggesting that reintegration changed predominantly within women across follow‐up periods. Despite the low ICC, mixed effects modeling remained appropriate because repeated observations from the same woman were not statistically independent.^34^, ^35^, ^36^

The fully adjusted model examined the association between continence status and social reintegration, adjusting for follow‐up time and baseline socio‐demographic, fistula‐related, and clinical covariates selected a priori based on clinical relevance and existing literature.^19^, ^35^ These included age, marital status, parity, province, fistula type, years lived with fistula, referral pathway, treatment hospital, surgical type, surgical difficulty, previous repair, postoperative complications, and fistula classification.

To assess whether the association between continence status and social reintegration changed over time, an interaction model including continence status and follow‐up time was fitted.^36^

Sensitivity analyses assessed potential facility‐level clustering using a three‐level mixed‐effects model with repeated observations nested within women and women nested within treatment facilities. Regression coefficients (*β*) represented mean differences in social reintegration scores, with statistical significance inferred when 95% CIs excluded zero.

## 3 RESULTS

The analytic cohort included 2172 women who underwent obstetric fistula repair, of whom 830 contributed at least one social reintegration assessment to the longitudinal analysis. The number assessed at each follow‐up period was 515 at baseline, 532 at 3 months, 572 at 6 months, and 549 at 12 months. Follow‐up attendance was non‐monotone for a substantial proportion of women, with some missing interim visits but returning for later assessments.

### 3.1 Participants' socio‐demographic information

Baseline participants' characteristics, measured at discharge after surgical repair, are presented in Table 1. Overall, 13.7% of the women remained incontinent, whereas 86.3% achieved continence. Among incontinent women at discharge, 63.4% were aged 35 years or older, compared with 43.2% of continent women. Most incontinent women (61%) had a parity of 1–3.

**TABLE 1:** Characteristics of participants according to their fistula continence status at baseline.

	Fistula continence status	
	Incontinent	Continent
	*N* = 262 (13.7%)	*N* = 1910 (86.3%)
*Women's age groups*		
<20	12 (5.6%)	101 (5.7%)
20–34	67 (31.0%)	913 (51.2%)
≥35	137 (63.4%)	770 (43.2%)
*Marital status*		
Not married	92 (39.5%)	468 (26.6%)
In union	141 (60.5%)	1292 (73.4%)
*Province in Zambia*		
Central + Lusaka + Western + Southern	28 (10.7%)	176 (9.3%)
Copperbelt + North‐western	3 (1.1%)	83 (4.4%)
Eastern	63 (24.0%)	580 (30.6%)
Luapula	71 (27.1%)	409 (21.6%)
Northern + Muchinga	97 (37.0%)	649 (34.2%)
*Parity level*		
0	6 (2.4%)	15 (0.8%)
1–3	155 (61.0%)	1048 (56.6%)
≥4	93 (36.6%)	787 (42.5%)
*Fistula type*		
Both/rectovaginal fistula	13 (5.0%)	357 (19.0%)
Vesicovaginal fistula	249 (95.0%)	1518 (81.0%)
*Years lived with fistula*		
<1 year	24 (10.2%)	353 (20.7%)
1–5 years	65 (29.4%)	541 (31.8%)
>5 years	132 (59.7%)	809 (47.5%)
*Fistula classification*		
Type I	20 (10.2%)	280 (23.0%)
Type II A(a)	14 (7.1%)	37 (3.0%)
Type II A(b)	64 (32.5%)	164 (13.4%)
Type II B(a)	30 (15.2%)	53 (4.3%)
Type II B(b)	25 (12.7%)	302 (24.8%)
Type III	44 (22.3%)	384 (31.5%)
*Had previous repairs*		
No	163 (72.1%)	1354 (87.1%)
Yes	63 (27.9%)	201 (12.9%)
*Client referral*		
Volunteers or self	230 (87.8%)	1751 (91.8%)
Health Facility & Fistula Foundation	32 (12.2%)	157 (8.2%)
*Treatment hospital*		
Kabwe + Monze + Chilenje	23 (8.8%)	201 (10.6%)
Northern region hospitals	122 (46.7%)	1041 (54.9%)
St. Francis	116 (44.4%)	653 (34.5%)
*Type of surgery*		
Others	10 (3.9%)	597 (31.4%)
VVF repair	249 (96.1%)	1305 (68.6%)
*Surgery difficulty*		
Simple	22 (9.8%)	762 (46.7%)
Intermediate	82 (36.4%)	646 (39.6%)
Difficult	121 (53.8%)	224 (13.7%)
*Post‐operative complications*		
None	215 (93.9%)	1592 (97.3%)
With complication	14 (6.1%)	45 (2.7%)

Furthermore, 95% of the incontinent women had vesicovaginal fistulas (VVF). Notably, 59.7% of the incontinent women had endured the condition for over 5 years before receiving surgical intervention. Difficult surgical repairs were more common among incontinent women (53.8%) than continent women (13.7%).

### 3.2 Social reintegration trajectories

Table 2 summarizes mean scores (0–5 scale) across the five social reintegration domains. Scores increased substantially by 3 months and plateaued thereafter.

**TABLE 2:** Obstetric fistula patients' mean social reintegration scores for each measure over time.

	Follow‐up time			
	Baseline	3 months	6 months	12 months
	Mean (95% CI)	Mean (95% CI)	Mean (95% CI)	Mean (95% CI)
Ability to socialize	2.20 (2.12, 2.28)	4.26 (4.18, 4.34)	4.24 (4.17, 4.31)	4.32 (4.25, 4.39)
Ability to work	2.30 (2.22, 2.37)	4.06 (3.98, 4.13)	4.15 (4.09, 4.22)	4.21 (4.15, 4.28)
Satisfaction with health	2.08 (2.02, 2.15)	3.89 (3.82, 3.96)	3.86 (3.79, 3.92)	4.04 (3.98, 4.10)
Satisfaction with life	2.12 (2.05, 2.19)	4.02 (3.94, 4.09)	4.05 (3.98, 4.12)	4.17 (4.10, 4.23)
Self‐esteem	3.04 (2.96, 3.13)	4.66 (4.60, 4.75)	4.67 (4.60, 4.73)	4.55 (4.47, 4.63)

*Note*: Each assessment uses a Likert scale of 1–5, with 1 being the worst and 5 the best.

### 3.3 Effects of obstetric fistula continence status and follow‐up time on social reintegration

The null mixed‐effects model yielded an ICC of <0.001, indicating minimal between‐patient variation (Table 3).

**TABLE 3:** Longitudinal associations between fistula continence status, follow‐up time, and social reintegration scores (0–100 scale).

	Model 1: Null model *β* ^a^ (95% CI)	Model 2: Full model *β* ^a^ (95% CI)	Model 3: Time‐interaction Model *β* ^a^ (95% CI)
Intercept	65.55 (64.46, 66.63)*	32.58 (23.32–41.83)*	29.21 (19.82–38.60)*
*Fistula continence status*			
Incontinent	–	Ref.	Ref.
Continent	–	38.62 (36.10–41.14)*	54.08 (50.92–57.25)*
*Time period (follow‐up)*			
Baseline	–	Ref.	Ref.
3 months	–	10.83 (8.23–13.43)*	–
6 months	–	9.34 (6.70–11.98)*	–
12 months	–	9.66 (6.97–12.34)*	–
Ordered follow‐up time trend	–	–	4.23 (3.06–5.39)*
*Age group*			
<20 years	–	Ref.	Ref.
20–34 years	–	−0.30 (−2.76–2.16)	−0.34 (−2.82–2.13)
≥35 years	–	−1.72 (−4.40–0.96)	−1.72 (−4.41–0.97)
*Marital status*			
Not married	–	Ref.	Ref.
In union	–	−0.56 (−1.84–0.71)	−0.72 (−2.00–0.56)
*Parity level*			
0	–	Ref.	Ref.
1–3	–	−2.15 (−10.08–5.79)	−2.52 (−10.48–5.45)
≥4	–	−1.43 (−9.32–6.45)	−1.74 (−9.66–6.18)
*Province*			
Central + Lusaka + Western + Southern	–	Ref.	Ref.
Copperbelt + North‐Western	–	0.89 (−2.75–4.53)	0.62 (−3.03–4.27)
Eastern	–	3.85 (0.16–7.54)*	3.57 (−0.15–7.29)
Luapula	–	−1.28 (−5.15–2.58)	−1.44 (−5.34–2.47)
Northern + Muchinga	–	−0.91 (−4.43–2.60)	−1.08 (−4.62–2.46)
*Fistula type*			
Other/both	–	Ref.	Ref.
Vesicovaginal	–	1.00 (−1.50–3.50)	0.86 (−1.65–3.36)
*Years lived with fistula*			
<1 year	–	Ref.	Ref.
1–5 years	–	1.16 (−0.55–2.87)	1.21 (−0.52–2.93)
>5 years	–	0.50 (−1.11–2.12)	0.61 (−1.02–2.24)
*Client referral*			
Volunteers/self	–	Ref.	Ref.
Health Facility & Fistula Foundation	–	−0.08 (−2.20–2.03)	−0.05 (−2.17–2.07)
*Treatment hospital*			
Kabwe + Monze + Chilenje	–	Ref.	Ref.
Northern region hospitals	–	2.42 (−0.95–5.79)	2.69 (−0.70–6.09)
St. Francis	–	2.32 (−1.13–5.77)	2.47 (−1.00–5.95)
*Surgery type*			
Other	–	Ref.	Ref.
VVF repair	–	−2.05 (−4.11–0.02)	−1.91 (−3.98–0.15)
*Previous repair*			
No	–	Ref.	Ref.
Yes	–	3.64 (1.37–5.92)*	3.86 (1.55–6.16)*
*Surgery complexity*			
Simple	–	Ref.	Ref.
Intermediate	–	−0.03 (−1.48–1.41)	0.03 (−1.42–1.47)
Difficult	–	−0.37 (−2.38–1.45)	−0.28 (−2.32–1.76)
*Fistula classification*			
Type I	–	Ref.	Ref.
Type II A(a)	–	0.06 (−3.63–3.75)	0.30 (−3.38–3.98)
Type II A(b)	–	0.32 (−1.88–2.53)	0.36 (−1.84–2.56)
Type II B(a)	–	0.00 (−3.06–3.07)	0.12 (−2.98–3.22)
Type II B(b)	–	0.18 (−1.85–2.21)	0.21 (−1.86–2.28)
Type III	–	0.69 (−1.31–2.82)	0.70 (−1.46–2.87)
*Post‐operative complication*			
None	–	Ref.	Ref.
Present	–	−1.10 (−5.13–2.93)	−0.88 (−4.93–3.17)
*Model diagnostics*			
Interaction (Continence status × Follow‐up time)	–	–	−4.85 (−6.30 to −3.40)*
Residual variance (*σ* ^2^)	612.0	122.0	123.6
Between‐patient variance (*τ* ^2^)	2.93 × 10^−9^	2.33 × 10^−9^	1.92 × 10^−8^
Intra‐class correlation (ICC)	<0.001	–	–

*Note*: Higher scores indicate better social reintegration.

^a^ *β* coefficients represent the mean differences in the social reintegration score (0–100).

* Statistically significant predictors and covariates.

In the fully adjusted mixed‐effects model, continence status after repair was strongly associated with social reintegration. Continent women had, on average, 38.62 points higher reintegration scores than incontinent women (*β* = 38.62; 95% CI: 36.10–41.14) on the 0–100 scale. Follow‐up time was positively associated with reintegration, with scores increasing by 10.83 points at 3 months (*β* = 10.83; 95% CI: 8.23–13.43), but almost plateaued at 9.34 points at 6 months (*β* = 9.34; 95% CI: 6.70–11.98) and 9.66 points at 12 months (*β* = 9.66; 95% CI: 6.97–12.34) relative to baseline. Eastern Province residence (*β* = 3.85; 95% CI: 0.16–7.54) and previous repair (*β* = 3.64; 95% CI: 1.37–5.92) were also positively associated with reintegration, whereas covariates were not.

In the time‐interaction model, continent women continued to report higher social reintegration scores (*β* = 54.08; 95% CI: 50.92–57.25) than their incontinent counterparts. Follow‐up time remained positively associated with social reintegration (*β* = 4.23; 95% CI: 3.06–5.39). However, the negative interaction between continence and time (*β* = −4.85; 95% CI: −6.30–−3.40) indicated that the difference between continent and incontinent women narrowed over time as reintegration scores among incontinent women gradually improved.

Sensitivity analyses supported the robustness of the findings. In the complete‐case analysis (710 observations from 271 women), continence remained strongly associated with higher reintegration scores (*β* = 32.37; 95% CI: 29.26–35.47), and follow‐up time remained positively associated with reintegration. The interaction between continence and time also remained negative (*β* = −6.28; 95% CI: −8.24 to −4.32), consistent with the multiply imputed model. A facility‐level clustering analysis similarly showed no meaningful change in effect estimates, supporting retention of the primary two‐level model.

**FIGURE 2:**
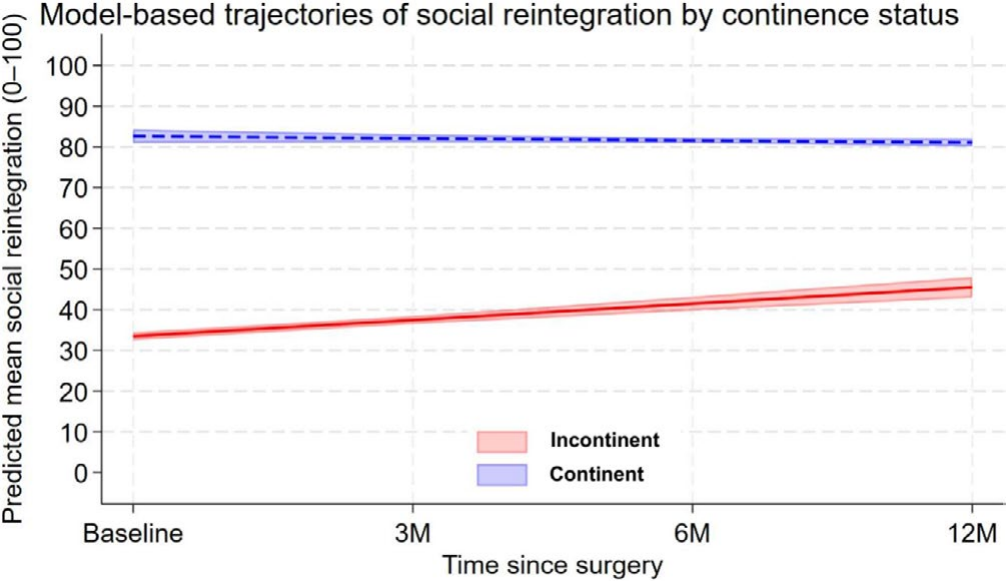
Model‐predicted social reintegration trajectories by continence status following obstetric fistula repair in Zambia.

Figure 2 illustrates model‐predicted social reintegration trajectories. Continent women maintained substantially higher predicted reintegration scores (approximately 82 points at 12 months) than incontinent women (approximately 45 points), although the difference narrowed over time.

## 4 DISCUSSION

This longitudinal study examined predictors and trajectories of social reintegration following obstetric fistula repair using a national clinical cohort in Zambia. Three findings are noteworthy. First, continence restoration was strongly associated with better social reintegration across all follow‐up periods. Second, the greatest improvements among incontinent women occurred within the first 3 months after surgery, after which differences with continent women narrowed. Third, most baseline socio‐demographic and clinical characteristics were not independently associated with reintegration after adjusting for continence status and follow‐up time. Together, these findings suggest that recovery after fistula repair is driven primarily by functional restoration and early postoperative adjustment rather than by baseline demographic differences.

The extremely low ICC (<0.001) suggests that variation in social reintegration occurred predominantly within women across follow‐up periods rather than between them. This pattern is consistent with the dynamic nature of recovery following fistula repair and supports the interpretation that reintegration evolves after surgery.^33^, ^36^

Restoration of continence emerged as the strongest predictor of social reintegration in women following fistula surgical repair. This finding is consistent with longstanding evidence that urinary and fecal incontinence disrupts women's economic participation through stigma, social isolation, marital dissolution, and economic marginalization.^4^, ^17^, ^37^ Successful surgical repair, therefore, removes a major barrier to resuming normal interactions and daily activities. Although this relationship is clinically intuitive,^34^, ^38^, ^39^ the present study contributes longitudinal evidence from a large national cohort, demonstrating both the magnitude and persistence of the association across repeated follow‐up periods in routine programmatic care. These findings also reinforce the importance of expanding access to quality fistula repair services in Zambia, where treatment remains centralized, and many women continue to face barriers to care.^14^, ^26^, ^34^, ^40^

The findings further identify the first 3 months after fistula surgery as an important period for postoperative recovery, regardless of continence status. Social reintegration improved markedly during this period, particularly among incontinent women, before stabilizing thereafter. Although psychosocial adaptation was not directly measured, previous studies suggest that women's social functioning often improves during early recovery.^38^, ^41^ Our findings indicate that structured follow‐up reintegration support during the first 3 months after surgery may complement clinical efforts in facilitating full recovery.

The narrowing difference in reintegration scores between continent and incontinent women over time suggests that recovery remains dynamic even among women with persistent symptoms. Although continent women consistently reported higher reintegration scores, incontinent women also showed gradual improvement. Qualitative studies have similarly described women rebuilding relationships, regaining social acceptance, and developing coping strategies after fistula repair.^18^, ^42^ Because psychosocial adaptation and social support were not directly measured, these factors should be interpreted as plausible contextual influences rather than confirmed explanatory mechanisms.

Most baseline socio‐demographic and clinical characteristics were not independently associated with social reintegration after adjustment for continence status and follow‐up time. This suggests that functional recovery following surgery may play a greater role in shaping women's participation and well‐being than baseline demographic or clinical characteristics.

Women residing in Eastern Province reported higher social reintegration scores, possibly reflecting differences in referral systems, community support, or reintegration services. Similarly, women with previous fistula repair had better social reintegration outcomes, which may reflect greater familiarity with the treatment process and recovery expectations. However, this could not be examined directly using the available clinical data.

The clinical profile of the cohort highlights broader challenges in fistula care in Zambia. Many women presented with complex fistula injuries, including urethral involvement, reflecting prolonged obstructed labor and potentially increasing iatrogenic injuries following cesarean section, as reported elsewhere in SSA.^17^ Although this study could not distinguish ischemic from iatrogenic fistulas, the findings underscore the importance of strengthening emergency obstetric care, surgical training, and access to skilled fistula treatment.

The relatively high continence rate observed in this cohort should be interpreted in the context of Zambia's specialized fistula care network, where trained surgeons, standardized protocols, outreach activities, and ongoing capacity building may contribute to improved surgical outcomes across facilities.^14^

This study provides one of the largest longitudinal analyses of social reintegration following obstetric fistula repair in SSA. By using repeated follow‐up data from multiple treatment centers across Zambia, the study offers a national perspective on reintegration trajectories. It identifies the first 3 postoperative months as an important period for recovery support. Application of the ICF framework further enabled an examination of recovery beyond surgical outcomes, including broader dimensions of participation and functioning.

Nevertheless, several limitations should be considered. First, the clinical dataset contained substantial missing data. Although multiple imputation and complete‐case sensitivity analyses yielded substantively consistent findings, the Missing at Random assumption could not be verified. Second, some clinical variables, including surgical difficulty, may reflect variability in clinical judgment across surgeons and facilities. Third, the database did not capture potentially important psychosocial factors such as stigma, social support, coping mechanisms, or household economic recovery, limiting our ability to examine mechanisms underlying reintegration. Finally, the data could not distinguish ischemic from iatrogenic fistulas or determine whether women with persistent incontinence subsequently underwent repeat surgery. Despite these limitations, the study provides important longitudinal evidence on social reintegration following fistula repair.

## 5 CONCLUSION

Achieving continence remains central to obstetric fistula recovery because of its strong association with improved social reintegration. However, the marked gains observed during the first 3 months after surgery suggest that early postoperative follow‐up and reintegration support may be important even for women with persistent symptoms. Integrating counseling, psychosocial support, and community reintegration approaches alongside surgical care may improve longer‐term recovery. These findings should be interpreted within the context of routine programmatic care and incomplete clinical data, and further research incorporating psychosocial and health system factors is warranted.

## AUTHOR CONTRIBUTIONS

Sianga Mutola led the conceptualization, formal analysis, data curation, and writing of the original draft. Bwalya Magawa Chomba, Nawi Ng, Menda M. Dhally, Valérie R. Louis, and Lowery Wilson Michael contributed to conceptualization, methodology, and critical review and editing of the manuscript. All authors contributed to the interpretation of the findings, provided substantial intellectual input, and approved the final version for publication.

## FUNDING INFORMATION

No funding was received for our study.

## CONFLICT OF INTEREST STATEMENT

The authors declare no conflict of interest.

## ETHICS STATEMENT

The FFTN database comprised routine clinical records and follow‐up questionnaires completed during patient care. The patients' data were collected by healthcare providers, including nurses and surgeons, as part of the hospitals' routine patient care and record‐keeping at admission and post‐operative follow‐up, in accordance with Zambia's Health Professions Act of 2009. This act requires healthcare providers to get parental or guardian consent for medical decisions involving patients under 18. We obtained ethical approval for using the FFTN database from the ERES Converge IRB (Ref. No. 2024‐Jul‐011) on August 11, 2023 and the University of Heidelberg Ethics Committee (Ref. No. S‐683/2024) on November 4, 2024. The datasets were fully pseudonymized by removing personal identifiers and assigning each patient a unique Fistula Foundation ID.

## Supporting information


**Supplementary material 1.** Community‐Based Identification, Screening, and Referral System for Women with Obstetric Fistula in Zambia (2017–2023), and the Obstetric Fistula Community‐Based Assessment Tool (OF‐COMBAT).

**Supplementary Material 2.** Social reintegration questionnaire used in the follow‐up of women after obstetric fistula repair.

**Supplementary Material 3** Detailed Statistical Analysis.

## Data Availability

The data that support the findings of this study are available on request from the corresponding author. The data are not publicly available due to privacy or ethical restrictions.
