# Supplementary material for: Social reintegration of women after obstetric fistula surgery: Evidence from a longitudinal multilevel mixed‐effects study in Zambia
Source: Acta Obstet Gynecol Scand. 2026 Jun 23;105(10):2048–59. doi: 10.1111/aogs.70296 (PMC13394903; doi:10.1111/aogs.70296)
Supplement: Supplementary file 1 [file AOGS-105-2048-s001.pdf]

Supplementary Material 3

Detailed Statistical Analysis

The FFTN database was recorded in Microsoft Excel and imported into Stata/SE 18 for Windows (StataCorp LLC, College Station, TX, USA) for cleaning and analysis, in accordance with the inclusion and exclusion criteria shown in Figure 1. Each record in the database represented a single surgical episode for an individual woman. Exclusion criteria were applied at the record level prior to analysis. A total of 404 records were excluded from the analytic dataset. Exclusion criteria included non-obstetric fistula diagnoses (n = 82), unrelated surgical diagnoses such as bladder stone or vaginal stenosis (n = 153), non-informative continence status at discharge (n = 134), procedures not relevant to obstetric fistula repair (n = 160), treatment outside Zambia (Mozambique) (n = 10), non-eligible postoperative complications (n = 19), and missing or non-informative incontinence status in the reintegration questionnaire (n = 68). Because some records met more than one exclusion criterion, category counts exceed the total number of excluded records. Therefore, the final analytical cohort consisted of 2,172 women who underwent obstetric fistula repair between 2017 and 2023. Social reintegration was assessed longitudinally, and each woman contributed repeated follow-up observations depending on attendance at scheduled assessments.

After confirming the absence of multicollinearity among the covariates, descriptive statistics were computed for participants' sociodemographic and clinical characteristics and summarized as column proportions. Furthermore, means and 95% confidence intervals (CI) were calculated for the components of the social reintegration scores across follow-up periods.

We assessed patterns of missing data and found that approximately 33% of cases were complete, whereas 67% had incomplete information across one or more variables, primarily for fistula classification (35%) and social reintegration score (22%). Missingness partly reflected irregular attendance at routine follow-up visits within the programmatic care. Among contributing longitudinal social reintegration data, 40.7% demonstrated non-monotone follow-up patterns, meaning they missed one or more interim visits but subsequently returned for later assessments. This pattern indicates that missingness did not exclusively reflect permanent dropout and supports the use of Multiple Imputation by Chained Equations (MICE) with 20 imputations to retain incomplete observations and reduce potential bias (1,2). The imputation included the outcome (social reintegration score), primary exposure (continence status), all covariates used in the analytical models, follow-up time, and treatment facility variables

associated with missingness. Missingness was examined against observed patient, clinical, and follow-up characteristics and appeared to be related to observed information rather than attributable solely to unobserved reintegration outcomes, supporting the plausibility – but not proof – of the Missing at Random (MAR) mechanism (1,2). Therefore, the findings should be interpreted cautiously, as the MAR assumption could not be empirically verified. To assess robustness, we additionally conducted a complete-case sensitivity analysis using the same model specifications on unimputed observations with complete data.

We then fitted multilevel mixed-effects linear regression models to the imputed data to examine the effects of continence status and follow-up time on social reintegration. Repeated measurements were nested within individual women because reintegration outcomes were assessed at baseline and at 3, 6, and 12 months. Therefore, the models included random intercepts at the patient level to account for correlation among repeated observations from the same individual and to appropriately model the hierarchical structure of the longitudinal data (3). Model assumptions were assessed using residual diagnostics, and no major deviations from linear model assumptions were observed.

We fitted an intercept-only (null) model to estimate variance components and calculate the intraclass correlation coefficient (ICC). The ICC was calculated as $\tau^2/(\tau^2 + \sigma^2)$, where τ^2 represents the between-patient variance and σ^2 the residual variance. The ICC from the null model was <0.001, indicating that only a negligible proportion of total variance in social reintegration scores was attributable to stable between-woman differences. This finding suggests that social reintegration changed predominantly among women across follow-up periods, rather than being driven by persistent individual differences. Such a pattern is plausible in the context of obstetric fistula recovery, where postoperative reintegration is expected to evolve dynamically over time following surgery and rehabilitation (4). Although a near-zero ICC suggests limited clustering at the individual level, multilevel mixed-effects modeling remained appropriate because repeated observations from the same woman were not statistically independent, and failure to account for this within-person correlation could lead to underestimation of standard errors and biased statistical inference (5). In the main, fully adjusted model, we examined the effect of continence on the social reintegration score, adjusting for follow-up time, which was included as a categorical variable to estimate differences at 3, 6, and 12 months relative to the baseline. This mixed-effects model also adjusted for baseline socio-demographic, fistula characteristics, and clinical confounders, including age, marital status, parity, province, fistula type, years lived with fistula, referral,

referral pathway, treatment hospital, surgical type, surgical difficulty, previous repair, postoperative complications, and fistula classification (6). The model specification followed an *a priori* analytical strategy informed by clinical relevance and existing literature, rather than automated variable selection procedures (7,8).

To evaluate whether the association between continence status and social reintegration changed over the follow-up period, we used a third model that included an interaction between follow-up time and continence status, along with all other covariates. This specification allowed assessment of a linear trend in the effect of continence across successive follow-up visits, consistent with recommended approaches for testing interactions involving ordered predictors such as time periods (5).

Finally, a sensitivity analysis assessed potential facility-level clustering using a three-level mixed-effects model with repeated observations nested within women and women nested within treatment facilities.

In all the models, the regression coefficients (β) represented mean differences in social reintegration scores, with positive coefficients indicating higher scores and negative coefficients indicating lower scores. Statistical significance was inferred when the 95% CIs did not include zero.

References

1. Van Buuren S. Flexible Imputation of Missing Data. 2nd ed. Boca Raton, FL: Chapman and Hall/CRC; 2018 July 17. DOI: 10.1201/9780429492259

2. Royston P, White I. Multiple Imputation by Chained Equations (MICE): Implementation in *Stata*. J Stat Soft. 2011;45(4). DOI: 10.18637/jss.v045.i04

3. Snijders TAB, Bosker RJ. Multilevel analysis: an introduction to basic and advanced multilevel modeling. 2. ed. Los Angeles, Calif.: SAGE; 2012.

4. El Ayadi AM, Painter CE, Delamou A, Barr-Walker J, Korn A, Obore S, et al. Rehabilitation and reintegration programming adjunct to female genital fistula surgery: A systematic scoping review. Intl J Gynecology & Obste. 2020;148(S1):42–58. DOI: 10.1002/ijgo.13039

5. Aiken LS, West SG, Reno RR. Multiple regression: testing and interpreting interactions. Reprinted. Newbury Park, Calif.: SAGE; 2010.

6. Goldstein H. Multilevel statistical models. 4th ed. Hoboken, N.J: Wiley; 2011. DOI: 10.1002/9780470973394

7. World Health Organization, editor. International classification of functioning, disability and health: ICF. Geneva: World Health Organization; 2001.

8. Harrell , FE. Regression Modeling Strategies: With Applications to Linear Models, Logistic and Ordinal Regression, and Survival Analysis. Cham: Springer International Publishing; 2015. (Springer Series in Statistics). DOI: 10.1007/978-3-319-19425-7
