# Supplementary material for: Social reintegration of women after obstetric fistula surgery: Evidence from a longitudinal multilevel mixed‐effects study in Zambia
Source: Acta Obstet Gynecol Scand. 2026 Jun 23;105(10):2048–59. doi: 10.1111/aogs.70296 (PMC13394903; doi:10.1111/aogs.70296)
Supplement: Supplementary file 2 [file AOGS-105-2048-s002.pdf]

Supplementary Material 2

Social reintegration questionnaire used in the follow-up of women after obstetric fistula repair

This questionnaire was administered to women who underwent obstetric fistula repair in Zambia as part of routine follow-up care within the Fistula Foundation Zambia treatment network. Assessments were conducted at discharge (baseline) and during scheduled follow-up visits at 3, 6, and 12 months after surgery.

The questionnaire comprised five domains reflecting functional and psychosocial aspects of recovery and social participation. Responses were recorded using standardized categorical scales, which were later converted to numerical scores and rescaled to a 0–100 social reintegration index for analysis.

Domain 1: Continence Status

Question:
What is your current continence status?

Response options:

1 – Dry

2 – Sometimes leaking

3 – Always leaking

Domain 2: Social Participation (Mix and Socialize)

Question:
Are you able to mix and socialize with other people in your community?

Response options:

1 – Strongly disagree

2 – Disagree

3 – Neutral

4 – Agree

5 – Strongly agree

Note: Some historical records include alternative coded wording such as “Somewhat,” reflecting earlier versions of the response scale. These responses were harmonized during data cleaning to maintain consistency across follow-up periods.

Domain 3: Ability to Work

Question:
Are you able to work or perform your usual daily activities?

Response options:

1 – Strongly disagree

2 – Disagree

3 – Neutral

4 – Agree

5 – Strongly agree

Domain 4: Life Satisfaction

Question:
Are you satisfied with your life?

Response options:

1 – Strongly disagree

2 – Disagree

3 – Neutral

4 – Agree

5 – Strongly agree

Domain 5: Health Satisfaction

Question:
Are you satisfied with your health?

Response options:

1 – Strongly disagree

2 – Disagree

3 – Neutral

4 – Agree

5 – Strongly agree

Domain 6: Self-Esteem

Question:
How do you feel about yourself?

Response options:

1 – Very low self-esteem

2 – Low self-esteem

3 – Neutral

4 – Good self-esteem

5 – Very good self-esteem

6 – Excellent self-esteem

This item was recorded numerically in the database and reflects perceived personal confidence and self-worth following surgical repair.
