# Supplementary material for: Social reintegration of women after obstetric fistula surgery: Evidence from a longitudinal multilevel mixed‐effects study in Zambia
Source: Acta Obstet Gynecol Scand. 2026 Jun 23:10.1111/aogs.70296. Online ahead of print. doi: 10.1111/aogs.70296 (PMC13394903; doi:10.1111/aogs.70296)
Supplement: Supplementary file 1 — Supplementary material 1. Community‐Based Identification, Screening, and Referral System for Women with Obstetric Fistula in Zambia (2017–2023), and the Obstetric Fistula Community‐Based Assessment Tool (OF‐COMBAT). [file AOGS-9999-0-s003.pdf]

Supplementary material 1

Community-Based Identification, Screening, and Referral System for Women with Obstetric Fistula in Zambia (2017–2023), and the Obstetric Fistula Community-Based Assessment Tool (OF-COMBAT)

1. Community-Based Identification and Outreach Activities (2017–2023)

Between 2017 and 2023, community-based activities were implemented through the Zambia Fistula Foundation Treatment Network (FFTN) to strengthen awareness, case identification, and referral of women with obstetric fistula. These activities were integrated within routine primary health care structures and relied on trained community volunteers and Safe Motherhood Action Groups (SMAGs) to conduct outreach and mobilization activities in communities.

Community outreach included structured sensitization meetings, distribution of informational and educational materials, radio programs, and engagement with community leaders and local stakeholders. These interventions were designed to reduce stigma, improve recognition of fistula symptoms, and facilitate timely referral to treatment centers. These activities contributed directly to patient identification and referral within the national treatment network.

Community leaders, including traditional and religious leaders, were engaged as gatekeepers to support community acceptance of fistula services and promote early health-seeking behavior among affected women. Health care providers at rural health facilities were also oriented on pre-referral assessment and referral procedures to ensure continuity of care across the referral pathway.

2. Patient Screening and Referral Process

The identification and referral of women with suspected obstetric fistula followed a structured continuum of care beginning at the community level and extending to specialized treatment centers.

Community volunteers conducted initial screening using a standardized verbal assessment tool. Women suspected of having an obstetric fistula were referred to nearby health facilities for clinical evaluation by trained health care providers. These providers assessed the patient’s symptoms and determined whether referral to a specialized treatment center was required.

The referral pathway typically followed the sequence:

Community identification

→ Screening using a standardized verbal tool

→ Health facility assessment

→ Referral to treatment center

→ Surgical repair

→ Postoperative follow-up and reintegration support

This referral system was designed to ensure continuity of care from community identification through treatment and long-term follow-up. The use of structured referral pathways was intended to improve patient linkage to care and reduce delays in accessing treatment services.

3. Description of the Obstetric Fistula Community-Based Assessment Tool (OF-COMBAT)

The Obstetric Fistula Community-Based Assessment Tool (OF-COMBAT) is a standardized verbal screening instrument used to identify women with symptoms suggestive of obstetric fistula at the community level.

The tool consists of 27 questions grouped into four categories based on a clinical screening framework. These categories assess clinical presentation, underlying causes, and the timing of symptom onset relative to childbirth. The tool is administered by trained community outreach workers and is designed to provide a tentative diagnosis that must be confirmed through facility-based clinical evaluation.

The screening framework in Annex 1, used with permission from the FFTN, includes:

Part I — Clinical presentation of vesico-vaginal fistula (VVF)

Part II — Clinical presentation of recto-vaginal fistula (RVF) or fourth-degree perineal tears

Part III — Causes of obstetric fistula

Part IV — Interval between childbirth and symptom onset

The diagnosis generated from the tool is considered provisional and requires confirmation through clinical testing, such as dye testing or other facility-based diagnostic procedures.

Appendix 1: OF-COMBAT Questionnaire and Scoring

OF-COMBAT Questionnaire and Scoring

Reliability

Split half reliability: *.72 to .86*　　　　Test retest reliability: *.79 to .93*

Test Scoring

Part I: Use this Section for Suspected VVF Cases

→ SKIP THIS SECTION if client does not describe leakage of urine and proceed to PART II.

VVF scores

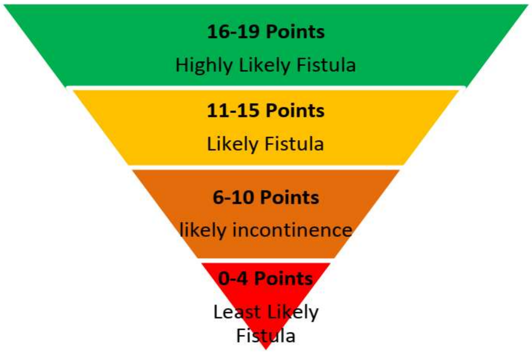

RVF scores

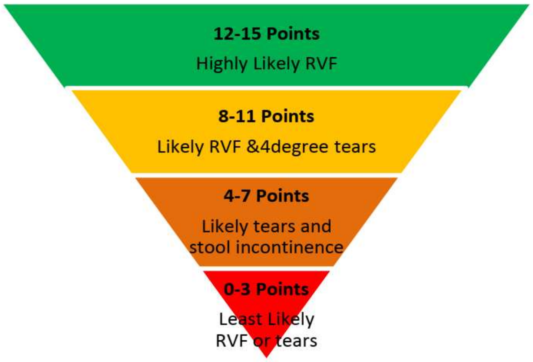

Part IA: Signs and symptoms (VVF). Instructions: For each question answered "Yes" score one (1) point. For each question answered "No" score zero (0) points.

	Yes (1)	No (0)
**1.** Do you continuously leak urine via your birth canal?		
**2.** Does the urine leak without you feeling it?		
**3.** Do you often find yourself wet with urine most if not all the time irrespective of where you are and what you are doing?		
**4.** Does the urine pass /leak on your bed while you are asleep most nights?		
**5.** Does the urine pass even when it's not provoked by anything?		
**6.** Do you need to use diapers and/or protective clothes to prevent being wet with the leakage?		
**Total Points Section IA:**		

Part IB: Validation Questions: (VVF). Instructions: For each question answered "Yes" score zero (0) points. For each question answered "No" score one (1) points.*

	Yes (0)	No (1)
**1.** Are there days or times that you are dry and not wet with urine?		
**2.** Do you ever have the urge to pass urine?		
**3.** Do you ever go to the bathroom/ washroom to pass urine?		
**4.** Do you wake up to pass urine at night/Are you able to get out of your bed to pass urine at night?		
**5.** Does urine leak when you cough or lift something?		
**6.** Can you go on with your work or leave the house without any diapers and/or protective clothing but still don't wet yourself?		
**Total Points Section IB:**		

****Scoring note**: When the respondent answers the same numeric question in Part A and B with identical response, the question becomes void and should be eliminated from the scoring. E.g., if the respondent answers 'yes' to question 1 in*

PART II: Use this section for Suspected RVF or perineal tear

→ SKIP THIS SECTION if client does not report leakage of stool and proceed to SECTION III.

Part IIA: Signs and Symptoms (RVF or perennial tear)

Instructions: For each question answered "Yes" score one (1) point. For each question answered "No" score zero (0) points.

	Yes (1)	No (0)
**1.** Do you pass stool via your birth canal?		
**2.** Do you pass gas with particles of stool via your birth canal?		
**3.** Do you have a visible tear between your vagina and your anal opening or connecting the two openings?		
**4.** Do you have stool incontinence that more often soils your birth canal?		
**Total Points Section IIA:**		

Part IIB: Validation Questions: (RVF or perineal tear)

Instructions: For each question answered "Yes" score zero (0) points. For each question answered "No" score one (1) points.

	Yes (0)	No (1)
**1.** Do you pass stool in the normal way without any problems?		
**2.** Do you pass gas well via your anal opening and your under pants always clean with no stool?		
**3.** Did you heal well after delivery and have discomfort, tears or injury around your birth canal or anal opening?		
**4.** When you having loss stool, Are you able get to the toilet and the stool comes out of your anal opening with not problem?		
**Total Points Section IIB:**		

****Scoring note**: When the respondent answers the same numeric question in Part A and B with identical response, the question becomes void and should be eliminated from the scoring. E.g. if the respondent answers 'yes' to question 1 in Part IIA and 'yes' to question 1 in Part IIB you should subtract this question from the scoring for Section IIA.*

PART III and PART IV: Use for BOTH Suspected VVF and RVF Cases

Part III: Causes of the obstetric fistula. Instructions: For each question answered "Yes" score one (1) point. For each question answered "No" score zero (0) points.

	Yes (1)	No (0)
1. Did you start leaking urine after delivery or Caesarean Section?		
2. Were you in labour for more than 48 hours?		
3. Did you deliver at home?		
4. Did you go to the health facility after more than 24 hours in labour?		
**Total Points Section III:**		

Part IV: The interval between the cause and effect

	Yes (1)	No (0)
1. Did the leaking start immediately after delivery?		
2. Did the leaking start within six weeks of delivery?		
3. Did the leaking start immediately after the catheter removal?		
**Total Points Section IV:**		

Final Scoring

**Scoring VVF (Part I, III, IV)**	
Section IA + IB (Max 12 points)	
Section III (Max 4 points)	
Section IV (Max 3 points)	
**Total VVF Score (Max 19 points)**	

**Scoring RVF (Part II, III, IV)**	
Section IIA + IIB (Max 8 points)	
Section III (Max 4 points)	
Section IV (Max 3 points)	
**Total RVF Score (Max 15 points)**	
